# Factors influencing specialist outreach and support services to rural populations in the Eden and Central Karoo districts of the Western Cape

**DOI:** 10.4102/phcfm.v7i1.750

**Published:** 2015-04-21

**Authors:** Johan Schoevers, Louis Jenkins

**Affiliations:** 1 Division of Family Medicine and Primary Health Care, Faculty of Health Sciences, Stellenbosch University, South Africa; 2 Family Physician, Mossel Bay subdistrict, Western Cape Department of Health, South Africa; 3 George Provincial Hospital, Eden District, Western Cape Department of Health, South Africa

## Abstract

**Background:**

Access to health care often depends on where one lives. Rural populations have significantly poorer health outcomes than their urban counterparts. Specialist outreach to rural communities is one way of improving access to care. A multifaceted style of outreach improves access and health outcomes, whilst a shifted outpatients style only improves access. In principle, stakeholders agree that specialist outreach and support (O&S) to rural populations is necessary. In practice, however, factors influence whether or not O&S reaches its goals, affecting sustainability.

**Aim and setting:**

Our aim was to better understand factors associated with the success or failure of specialist O&S to rural populations in the Eden and Central Karoo districts in the Western Cape.

**Methods:**

An anonymous parallel three-stage Delphi process was followed to obtain consensus in a specialist and district hospital panel.

**Results:**

Twenty eight specialist and 31 district hospital experts were invited, with response rates of 60.7% – 71.4% and 58.1% – 74.2% respectively across the three rounds. Relationships, communication and planning were found to be factors feeding into a service delivery versus capacity building tension, which affects the efficiency of O&S. The success of the O&S programme is dependent on a site-specific model that is acceptable to both the outreaching specialists and the hosting district hospital.

**Conclusion:**

Good communication, constructive feedback and improved planning may improve relationships and efficiency, which might lead to a more sustainable and mutually beneficial O&S system.

## Abstrait

**Facteurs influençant l'extension des services spécialisés et de soutien aux populations rurales dans les districts d'Eden et du Central Karoo du Western Cape**.

**Contexte:**

L'accès aux soins de santé dépend souvent de l'endroit où l'on vit. Les populations rurales ont un bilan de santé plus négatif que leurs homologues urbains. L'extension des services spécialisés aux communautés rurales est une manière d'améliorer l'accès aux soins. Un style de rayonnement à multiples facettes améliore l'accessibilité et l’état de santé, alors que le déplacement des patients externes ne fait qu'améliorer l'accès. En principe, les parties prenantes sont d'accord que l'extension des services spécialisés et de soutien (O&S) aux populations rurales est nécessaire. Cependant, dans la pratique, certains facteurs déterminent si les O&S atteignent ou non leur objectif, et affectent leur durabilité.

**Objectif et cadre:**

Notre but était de mieux comprendre les facteurs qui contribuent au succès ou à l’échec de l'extension des O&S aux populations rurales du districts de l'Eden et du Central Karoo du Western Cape.

**Méthodes:**

On a suivi un processus Delphi anonyme en trois étapes pour obtenir un consensus dans un comité de spécialistes et d'experts d'hôpitaux de district.

**Résultats:**

On a invité huit spécialistes et 31 experts d'hôpitaux de district, avec des taux de réponse de 60.7% – 71.4% et 58.1% – 74.2% respectivement au cours des trois étapes. Ils ont trouvé que les relations, la communication et la planification étaient des facteurs ayant une incidence sur la prestation de service par rapport aux tensions du développement des capacités, qui affecte l'efficacité des O&S. Le succès du programme des O&S dépend d'un modèle adapté au site qui soit acceptable aux spécialistes de l'aide et à l'hôpital de district qui les reçoit.

**Conclusion:**

Une bonne communication, des commentaires constructifs et une meilleure planification pourront améliorer les relations et l'efficacité, qui produiront un système d'O&S plus durable et mutuellement bénéfique.

## Background

Access to health care, like childhood survival, often depends on where one lives.^1^ The infant mortality rate in rural South Africa (SA) is 52.6 per 1000 births, compared to 32.6 per 1000 births in urban areas.^2^ Furthermore, three of the four districts in SA with the highest HIV prevalence are rural.^3^ These being two commonly used health indicators, it is clear that rural populations have significantly poorer health outcomes than their urban counterparts.

About half of the world's population lives outside major urban centres, where health services and specialist medical services are concentrated.^4^ Rural SA is home to 43.6% of the population, but is served by only 12% of doctors and 19% of nurses.^2^ Of the 1200 medical students graduating in SA annually, only about 35 work in rural areas in the long-term.^2^ There are 30 generalists and 30 specialists/100 000 people in urban areas, compared to an average of 13 generalists and 2 specialists/100 000 people in rural areas.^5^ The question arises whether the poorer access to particularly specialist services is one of the contributing factors towards poorer outcomes.

Stakeholders agree that specialist outreach and support (O&S) to rural communities is necessary, as it improves access to specialised healthcare services, effectiveness, efficiency, and relationships between the different levels of health care.^1,6,7^ In practice, however, there are many factors that influence whether or not O&S reaches its goals, which in turn affects the sustainability of O&S projects. Understanding these factors would aid recommendations for a suitable model for O&S.

Shifted outpatient styles of outreach, where the outreaching specialist merely sees patients without focusing on skills transfer and engaging with local health carers, focus only on service delivery and improved access, but do not impact health outcomes.^7^ A multifaceted outreach service that focuses on capacity building as well as service delivery improves outcomes and efficiency, whilst reducing use of inpatient services.^7^ For the purposes of this study O&S referred to a multifaceted outreach service. Capacity building includes the transfer of knowledge and skills, as well as developing and maintaining codependant support systems between district and regional healthcare systems.

O&S reduces cost to the patient by 19%, and also reduces time wasted by the patient.^7^ It increases attendance of booked appointments and patient satisfaction, and leads to more guideline-consistent care. It is unclear whether outreach reduces radiology and laboratory costs, but it reduces outpatient treatment modalities and admissions for inpatient treatment.^7^ Although O&S is more costly than hospital-based care, multifaceted outreach interventions improve health outcomes, which justifies its use.^7^

Most research on specialist outreach has been done in urban settings using the shifted outpatients model, where the benefits were few.^7^ There is little available research on the effect of specialist outreach to rural communities, where greater benefit is expected.^7^

Specialists’ opinion towards outreach differs, some criticising inefficient use of scarce specialist resources, others praising its effectiveness.^7^ Many healthcare providers fail to appreciate that health care is delivered within a mutually dependant system. Specialists are dependent on a functional primary care service to protect them from inappropriate problems and to provide a step-down facility in order to allow them to meet their objectives. Developing and strengthening primary care services is a critical step in securing accessible specialist services. A close relationship between components of the health system and a well-functioning referral system with clear referral criteria are the key to achieving equity in access to appropriate levels of care. There also needs to be a shift from a movement of patients to the movement of capacity and resources within the health system.^1^

Outreach that is sustainable, properly organised, relevant to local needs and has an adequate specialist base can integrate and support secondary and primary health care, thus benefitting rural communities.^7^ Poorly planned and conducted outreach can draw resources away from primary health care.^7,8^

In the Western Cape the primary objective of outreach is to ensure that patient care is of the highest quality within the available resources.^6^ Responsibilities of visiting specialists in SA include ward rounds, outpatient clinics, surgical procedures, morbidity and mortality meetings and other measures to evaluate quality of care, educational meetings, and developing guidelines and protocols with in-service training on these.^6,9,10^ Other responsibilities that can be included are professional and/or personal and managerial support.^1,9,10^

In the Eden and Central Karoo districts of the Western Cape of SA there are one level 2 (regional) hospital and 10 level 1 (district) hospitals. All clinical disciplines carry out outreach, with varying frequencies. On average the 4 main district hospitals receive 17 specialist outreach visits per month, whilst the smaller district hospitals receive 3 specialist visits per month, as per interviews with the respective clinical managers. A typical outreach visit includes a problem ward round, outpatient clinic, theatre list for some surgical disciplines, and formal or informal educational sessions.

O&S services in rural SA should focus on empowerment and relationship building with local doctors, rather than service delivery. They should be regular, sustainable and linked to continual professional development.^11^ Problems commonly encountered by specialists are poor planning, rapid turnover of district hospital staff, unavailability of essential equipment or drugs, and inadequate preparation of patients for surgery. Resistance to change and limited teaching opportunities due to work pressure or indifference are also problematic.^11^ As the district hospital work has to continue despite the specialist visit, O&S can create tension between service and teaching needs. Specialists are sometimes unaware of this disruption and have unreasonable demands.^11^

It has been recommended that specialists doing outreach should have the correct attitude and be able to adapt to rural conditions, without compromising essentials.^11^ The same specialist should visit a specific hospital on a regular basis. Teaching should focus on common conditions. Protocols for managing these conditions should be established in consultation with the district hospital management. Surgeons should consider the peri-operative limitations in rural hospitals and confine surgery to what local doctors can be taught to do. Furthermore, a dedicated district hospital doctor should coordinate the local practicalities and follow-up of patients seen. The rural hospital also needs to rearrange its schedule and staff for the day of outreach.^11^

### Aim and objectives

There is little research on the attitudes of stakeholders in the Western Cape towards specialist O&S. The aim of this study was to better understand factors associated with the success or failure of specialist O&S services to rural populations in the Western Cape. The objectives included reaching consensus between outreaching specialists, and between rural district hospital doctors on the major factors influencing O&S services, and making recommendations for provision of O&S services to rural populations.

## Research methods and design

### Study design

The Delphi method was used to obtain consensus.^12^ Specialists and district hospital doctors and nurses were asked to give their opinion on the major factors influencing O&S.

### Setting

**FIGURE 1:**
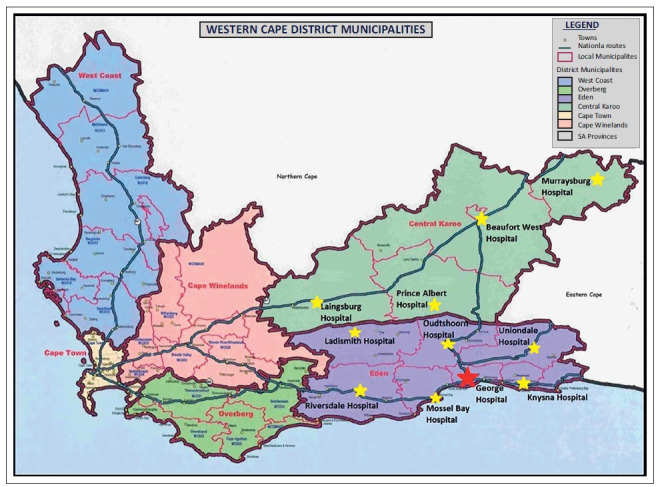
Map indicating Eden and Central Karoo districts in the Western Cape.

Figure 1 shows the Eden and Central Karoo districts, which cover an area of 61 573 km^2^,with an estimated population of 569 536.^13^ They are serviced by one regional hospital in George, and 10 district hospitals of varying sizes. All the hospitals are accessible by tarred road; the furthest from George is Murraysburg Provincial Hospital (327 km).

### Study population and sampling strategy

All public service specialists and specialised medical officers currently and previously involved in O&S in the two districts were invited to participate, comprising the specialist panel. Specialised medical officers refer to career medical officers in a specific department who are trusted by their heads of departments to conduct O&S visits. All hospital medical and clinical managers, as well as some career medical officers or nurses actively involved in O&S at Mossel Bay, Oudtshoorn, Knysna, Riversdale, Ladysmith, Beaufort West and Prince Albert district hospitals were invited to participate, comprising the district hospital panel. Panels bigger than 30 members have not been shown to improve results.^12^ Informed consent was obtained from all study participants.

Only O&S from level two to level one was evaluated. Outreach activities from level three to level two, by non-medical personnel, and by private sessional specialists were excluded.

### Data collection

Questionnaires were developed from the literature and input from local and national experts, including members of the local O&S service, district healthcare management and academics with a special interest in O&S. Experts were consulted via telephonic interviews or email on the major factors influencing O&S services. An anonymous parallel three-stage Delphi process was followed to obtain consensus.

Data were collected by sending and retrieving questionnaires via email or fax from May to July 2012. Consensus was defined as 70% of panel members giving the same response to a statement. Statements were made regarding O&S and panel members were asked to respond to these using a Likert scale, with the following options: Agree strongly, Agree, Disagree and Disagree strongly. A neutral middle option was excluded to force panel members to choose either a positive or negative option. Panel members were also given the option to comment on each statement, and to give qualitative feedback regarding other issues affecting O&S that were not covered by the statements. Statements where consensus was reached were removed from subsequent rounds.

### Data analysis

Nominal and ordinal data were converted into simple descriptive statistics, in consultation with the University of Stellenbosch's Centre for Statistical Consultation.

### Ethical considerations

Ethics approval was granted by the Human Research: Ethics Committee of the University of Stellenbosch (Ref. No.: S11/11/023). Permission was granted by the Research Committee of the Department of Health of the Western Cape, and district hospital management.

## Results

Twenty eight experts were invited to the specialist panel, and 31 to the district hospital panel. The distribution of experts between the different specialist departments and district hospitals, as well as the response rates for each round, are shown in Table 1a and Table 1b.

**TABLE 1a:** Panel composition and response rates.

Composition		Number invited (currently and previously involved in O&S)	Round 1 response	Round 2 response	Round 3 response
Specialist departments	Number currently involved in O&S				
Anaesthetics	2	2	1	1	1
Family Medicine	3	3	3	1	3
General Surgery	3	4	1	1	1
Internal Medicine	4	4	4	4	4
Obstetrics/Gynaecology	3	3	3	3	3
Ophthalmology	3	2	0	0	0
Orthopaedic Surgery	3	3	3	3	3
Paediatrics	4	4	2	2	3
Psychiatry	2	3	2	2	2
**Total**	**27**	**28**	**19 (67.9%)**	**17 (60.7%)**	**20 (71.4%)**

O&S, outreach and support.

**TABLE 1b:** Panel composition and response rates.

District hospitals	Number invited	Round 1 response	Round 2 response	Round 3 response
Beaufort West	3	1	1	1
Knysna	5	5	4	5
Ladysmith	3	3	3	3
Mossel Bay	7	5	5	6
Oudtshoorn	6	4	2	5
Prince Albert	3	2	1	2
Riversdale	4	2	2	1
**Total**	**31**	**22 (71.0%)**	**18 (58.1%)**	**23 (74.2%)**

O&S, outreach and support.

†, Indicate where a statement was not presented to a panel/where consensus was not reached in one of the panels, and the statements in bold were deemed key findings.

### Round 1

Fifty six and 50 statements were evaluated by the specialist panel and district hospital panel respectively. Consensus was reached on 8 statements in the specialist panel, and on 6 statements in the district hospital panel. These statements were removed from subsequent rounds. Panel members had the opportunity to comment on the statements or to suggest additional issues that needed to be explored. The remaining statements where consensus was not reached, as well as new modified statements, were transferred to round two.

### Round 2

The specialist panel evaluated 54 statements during round 2. These included the statements from round one where consensus was not reached, as well as 4 new statements that were based on comments during round one, and 3 confusing statements that were modified and/or expanded to 5 new statements. Consensus was reached on 29 of these statements during round 2.

The district hospital panel evaluated 49 statements during round 2. These included the statements from round one where consensus were not reached, as well as 2 new statements that were based on comments during round one, and 3 confusing statements that were modified/expanded to 5 new statements. Consensus was reached on 33 of these statements during round 2.

### Round 3

During round 3 the options on the Likert scale were reduced to only ‘Agree’ and ‘Disagree’. The remaining 25 statements in the specialist panel were evaluated, and consensus was reached on 18 statements. In the district hospital panel 15 statements were evaluated and consensus was reached on 5 statements.

In total consensus was reached on 55 of the 62 statements in the specialist panel and on 44 of the 54 statements in the district hospital panel (Table 2).

**TABLE 2:** Statements where consensus was reached.†

Statements	Specialists	District hospitals
There is enough regional hospital management support for O&S.	Agree	Agree
There is enough district hospital management support for O&S.	Agree	Agree
The planned O&S for the week/month is discussed with all the involved staff in the specialist department.	Agree	–
The planned O&S for the week/month is discussed with all the involved staff at the district hospital.	–	Disagree
Inefficient travel arrangements (like transport, meals, accommodation) are barriers to O&S that happen frequently.	Disagree	–
Travel arrangements (transport, meals and accommodation) are the responsibility of the district hospital.	–	Disagree
O&S visits can be scheduled better to disrupt district hospital less.	Agree	Agree
If O&S visits are cancelled, it is with sufficient warning.	–	Disagree
Both the specialist and district hospital should reflect on O&S encounters in a regular written report.	Agree	Agree
O&S clinics are overbooked.	Disagree	–
Overbooking can be overcome by appropriate referrals and work-up.	Agree	–
Overbooked clinics are due to the number of patients needing specialist care.	–	Agree
There is a need for more O&S visits.	–	Agree
Appropriate patients are seen during O&S.	Agree	–
Patients seen are over-investigated prior to O&S.	Disagree	–
O&S leads to fewer referrals to the regional hospital.	Agree	Strongly agree
A call the day before an O&S visit to inform of the number of booked patients would be helpful.	Agree	–
Patients must be discussed with the specific specialist at booking of the patient.	Agree	Agree
Preferred method.	Email	Telephone
O&S can be more useful with more email/Skype/cell phone/teleconferencing.	Agree	Agree
O&S leads to more efficient patient care.	Strongly agree	Strongly agree
Patients seen on O&S get the same standard of care as at the specialist's base hospital.	Agree	Agree
There are enough district hospital doctors to make O&S work.	Disagree	Disagree
There are enough specialists to make O&S work.	–	Agree
Smaller hospitals generally find it more difficult to live up to the specialist's expectations.	Disagree	–
Sessional specialists should also be involved in O&S.	Agree	Agree
Allied health professionals like specialist nurses, sonographers, etc. should also be involved in O&S.	Agree	Agree
The main focus of O&S currently is service delivery.	Agree	–
The main focus of O&S should be capacity building.	Agree	Agree
A district hospital doctor is present during most consultations.	Disagree	–
Better scheduling of the day's work can allow a district hospital doctor to be present during most consultations.	–	Agree
A district hospital doctor present during consultation will improve O&S.	Strongly agree	Strongly agree
Doctors working in outlying primary health care clinics should also attend O&S sessions, regardless of the logistical challenges.	Agree	–
Logistical support and capacity building should be equally important reasons to have a district hospital doctor present during consultations.	Agree	Agree
It is essential for surgical specialities to do surgery whilst on O&S.	Agree	Agree
Surgery done should be aimed towards what district hospital doctors can be taught to do safely.	Agree	Agree
Anaesthetic and postoperative care are always considered prior to booking patients for surgery.	Agree	Agree
Patients for surgery are properly prepared.	Agree	–
The necessary equipment is available for surgery.	Agree	–
Patients should only be booked for theatre after discussion with the surgeon or being seen by the surgeon.	Agree	–
Most of the expected investigations are available at district hospital level.	Agree	–
Medications prescribed by specialists are generally available at the district hospital.	Agree	Agree
A ward round seeing problem patients as well as random patients will be more helpful than seeing only problem patients.	Agree	Agree
Protocols for the management of common conditions are available.	Agree	Agree
The above protocols are helpful.	–	Agree
Patients seen during O&S are generally sorted out sooner.	Agree	–
Specialists regularly attend morbidity and mortality meetings at district hospital.	Disagree	Disagree
The morbidity and mortality meetings influence quality of care.	Agree	Agree
A specific specialist should be connected to a specific district hospital.	Agree	Agree
Most specialists have the correct personality for O&S (i.e. attitude, motivation, adaptability).	Agree	–
Exchange between district hospital doctors and regional hospital doctors for one/two weeks will aid understanding of each other's context, etc.	Strongly agree	Agree
A district-wide clinical day once or twice a year, for regional and district doctors to interact, will be good to share clinical and operational experiences.	Agree	Agree
O&S leads to easier referral up and down the referral chain due to better relationships.	Strongly agree	Agree
Constructive feedback on the quality of all referrals to specialist care will be helpful.	Agree	Strongly agree
Mentoring district hospital doctors in professional issues is part of O&S.	Agree	Agree
There is a dedicated educational session during O&S.	Agree	Agree
These sessions are well attended.	Agree	–
Statements	Specialists	District hospitals
Educational sessions are relevant to district hospitals.	–	Agree
Topics for educational sessions are known well in advance.	Disagree	Disagree
District hospital doctors are generally open to advice and change.	Agree	–
District hospital doctors are generally committed to O&S.	Disagree	–
Outreaching specialists are generally committed to O&S.	–	Agree
District hospital doctors have unrealistic expectations of O&S.	Agree	–
Specialists have unrealistic expectations of O&S.	–	Disagree
The success of O&S is context-/site-specific.	Agree	–
O&S is satisfying.	Agree	Agree
Educational sessions are relevant to district hospitals.	–	Agree
Topics for educational sessions are known well in advance.	Disagree	Disagree
District hospital doctors are generally open to advice and change.	Agree	–
District hospital doctors are generally committed to O&S.	Disagree	–
Outreaching specialists are generally committed to O&S.	–	Agree
District hospital doctors have unrealistic expectations of O&S.	Agree	–
Specialists have unrealistic expectations of O&S.	–	Disagree
The success of O&S is context-/site-specific.	Agree	–
O&S is satisfying.	Agree	Agree

O&S, outreach and support.

†, Indicate where a statement was not presented to a panel/where consensus was not reached in one of the panels, and the statements in bold were deemed key findings.

No consensus was reached on 7 statements in the specialist panel, and 10 statements in the district hospital panel (Table 3).

**TABLE 3:** Statements where consensus was not reached.

Specialists	District hospitals
The main focus of O&S currently is capacity building.	The main focus of O&S currently is capacity building.
The main focus of O&S should be service delivery.	The main focus of O&S should be service delivery.
–	The main focus of O&S currently is service delivery.
There is a dedicated liaison doctor at the district hospital to coordinate the O&S visit.	There is a dedicated liaison doctor at the district hospital to coordinate the O&S visit.
–	District hospitals have a long-term roster of O&S dates.
The referral letters are adequate if no district hospital doctor is present.	Referral letters from regional hospitals to district hospitals are generally adequate.
–	It is almost impossible for doctors working in outlying primary healthcare clinics to attend O&S sessions.
Protocols are generally followed.	Patients are referred according to the protocols without problems.
–	Most specialists have the correct personality for O&S (i.e. attitude, motivation, adaptability).
–	If O&S visits are cancelled, patients are accommodated by an extra visit or during the next O&S.
There are enough specialists at regional hospital to make O&S work.	–
Patients seen during O&S are not worked up appropriately.	–

O&S, outreach and support.

## Discussion

**FIGURE 2:**
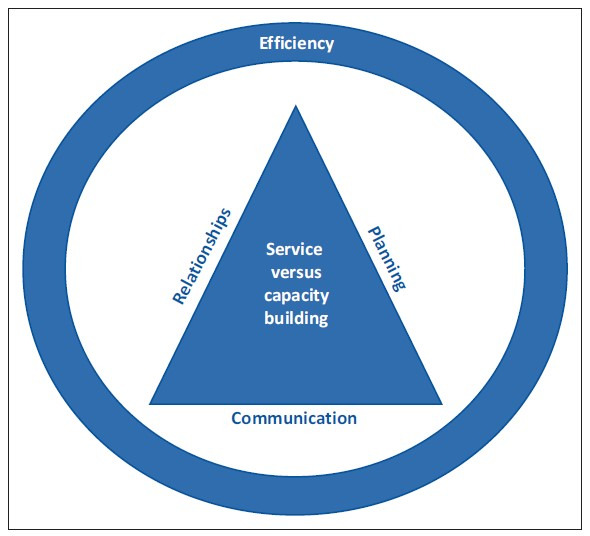
Outreach themes.

The key findings, which appear in bold in Tables 2 and 3, can be grouped together in the following themes, and are interconnected to a greater or lesser degree, as shown in Figure 2: relationships; communication; planning; service vs capacity building; and efficiency.

Relationships and communication are central themes in O&S programmes.^1^ Comments from the questionnaires that summarised this well were ‘It's all about relationships,’ and ‘Communication is the key’.

Although O&S is part of the job description of regional specialists, it only works in an efficient way if the relational (and emotional) component of this service is recognised and prioritised.^6^ O&S generally improves relationships, but O&S that is not properly planned can draw resources away from primary health care and could lead to intense frustration for the specialists or district hospital staff.^7^ Specialists and district hospital staff should be equal partners in this relationship. It appears that specialists are generally committed to O&S and are actively involved; however, the commitment of district hospital staff towards O&S is questioned by the specialist group. Without building mutually beneficial and codependant relationships, O&S programmes are bound to fail, paradoxically making specialist care more inaccessible for rural patients. Consensus was reached by both panels on ways to develop healthy relationships and communication: through constructive feedback on referrals, mutual reporting on outreach, mentoring by specialists, and a combined clinical day for specialists and district doctors once or twice a year.

Communication can improve efficiency of O&S in various ways. Both groups agreed that patients should be discussed with the outreaching specialist when an appointment is made for them.^10^ Having a specific specialist visiting a specific district hospital makes this a lot easier.^11^ The specialists preferred the discussion to be email-based, whilst the district hospital group preferred it to be telephone-based. Reasons mentioned for email-based discussions were to keep medico-legal records and to limit interruptions during consultations. Email-based discussions naturally involve the use of technology (Internet connection or smartphone), and this is not always available; however, fax-to-email is a relatively simple solution to this problem. Reasons mentioned for telephonic discussions included them being easier, quicker, and limiting delayed and/or non-responders.

These discussions serve many purposes. Many of the patients can be managed without the specialist even seeing the patient. It also leads to fewer inappropriate referrals or ‘dumping’ and to more appropriate work-up of patients, which leads to less overbooked O&S clinics. It also serves as a learning opportunity, and should be seen as part of capacity building. Patients consulted this way are ensured longitudinal care by the doctors involved, whilst the district hospital doctors are given the opportunity to develop capacity and the specialist is given logistical support by the district hospital doctors.

There was consensus that O&S is context- or site-specific. Comments from participants suggest that the same district hospital might have different O&S experiences, depending on the specific specialist or specialty. An exciting prospect is the apparent openness to exchange doctors between district and regional hospitals for a week or two at a time. This will serve the dual purpose of aiding understanding of each other's context, as well as giving doctors the opportunity to learn from each other. This will involve careful planning by mid-level and senior members of staff from both teams.

Whilst the focus of O&S currently seems to be service delivery, most participants agreed that capacity building should rather be the focus.^11^ Many participants commented that the focus should be 50% on service delivery and 50% on capacity building. If the O&S programme in the Eden and Central Karoo districts could make the shift from a service delivery/shifted outpatients model to a multifaceted/capacity building model, the health outcomes of patients in these rural districts could hopefully improve, as suggested in the literature.^7^

Capacity building should not only be limited to traditional lectures, but should be expanded to include bedside teaching, case-based discussions, teaching of procedural skills and mentorship in professional issues.^9,10^ In order to do this there needs to be a district hospital doctor present for most of the outreach visit. Both panels agreed that having a district hospital doctor present during consultations would improve O&S. A reason often mentioned for the inability to do this is the other responsibilities that the district hospital doctors have outside of the O&S visits, and the disruption that the O&S visit causes to the routine functioning of the district hospital.^11^ One of the participants summarised the counter-argument to this well: ‘O&S should be part of the basic core function of a district hospital and therefore cannot be seen as a disruption.’

One should be cognisant of the fact that O&S causes disruption in the regional hospital as well as in the district hospitals, especially when more than one outreaching specialist visits a district hospital on the same day. Both panels agreed that the district hospital doctor presence could be improved by better scheduling of O&S visits, as well as better planning of the day's work at the district hospital.^11^

An alarming fact was that the district hospitals agreed that the planned O&S visits for the week or month are not discussed with the staff involved. This could be due to the fact that there might not be a dedicated liaison doctor at the district hospital to coordinate the visit. With improved planning and/or communication, this could be improved. If there is a long-term programme available for O&S visits and this is adhered to, the workload could be shifted to free up doctors on those days.^11^

O&S visits should preferably be confined to one specialist per hospital per day, to avoid overwhelming the capacity of the district hospital. Human resources or the lack thereof was another reason often mentioned for the lack of a district hospital doctor presence. It seems as if there are not enough district hospital doctors to make O&S work properly. Whilst it was mentioned in the district hospital group that smaller district hospitals often find it more difficult to meet the specialist's expectations, the specialists remarked that some smaller hospitals often perform the best. As it is unlikely that district hospitals will receive extra posts to make O&S work, the old cliché of working smarter instead of harder seems to ring true in this instance.

### Recommendations

The success of the O&S programme is dependent on a model that is acceptable to both the outreaching specialists and the hosting district hospital. These two groups should agree on an appropriate model that is well planned, communicated to all involved staff, and adhered to. If O&S visits are seen as a basic function of a district hospital and not as a disruption and/or intrusion, the perceived attitudes towards commitment might change for the better. It is therefore recommended that:

- The main focus of O&S should be capacity building, involving most staff at some stage.
- Both the specialists’ and district hospitals’ service commitments and constraints should be respected in the scheduling of O&S visits.
- A long-term roster for O&S should be distributed to all involved staff.
- The O&S for the week or month should be discussed with all involved staff in the specialist department and district hospital by a dedicated person.
- A specific specialist should be linked to a specific district hospital for an agreed period.
- Patients should be discussed with the specific specialist prior to making an appointment – preferably via email unless urgent, logistically impossible or agreed beforehand.
- Specialists should respond promptly to these discussions.
- O&S visits should be limited to one specialist per district hospital per day, according to what the district hospital can handle.
- The district hospital workload should be managed to enable doctors to present their patients.
- Logistical support and capacity building should be equally important reasons for district hospital doctor presence.
- Professional relationship building and mentorship should form an integral part of the O&S visit.
- Issues that could be explored in future are:
  ■ Seeing random inpatients as well as problem patients.
  ■ Exchange programmes between the district and regional hospitals.
  ■ Constructive feedback from all referrals to the regional hospital.
  ■ Involving sessional specialists, and allied health professions that are not available at the district hospital, in O&S.

A report of the results was presented to the two panels and district health and regional hospital management, with a proposed model for O&S.

### Limitations

This study was conducted in the Western Cape, which has the highest number of doctors per capita in SA.^14^ Human resources influence the way in which O&S happens, and provinces with fewer doctors might struggle to implement the above recommendations.

Due to the relatively small pool of experts available, numerous participants from the different specialist departments and district hospitals were invited. Cross-contamination of ideas were therefore possible, due to close working relationships.

Not all statements were examined by both groups. Statements where the one group directly evaluated an issue regarding the other group were not necessarily evaluated by the latter.

## Conclusion

Providing O&S to rural populations remains an integral part of improving access to specialist care for rural populations. A multifaceted style of outreach remains the most effective way of providing O&S services. Due to the complex interpersonal and interprofessional dynamics between the involved parties, it is likely that there will always be the potential for conflict. With good communication, constructive feedback and improved planning, relationships and efficiency may improve, which might lead to a more sustainable and mutually beneficial O&S system.
